# Supplementary material for: VESPER: global and local cryo-EM map alignment using local density vectors
Source: Nat Commun. 2021 Apr 7;12:2090. doi: 10.1038/s41467-021-22401-y (PMC8027200; doi:10.1038/s41467-021-22401-y)
Supplement: Supplementary file 1 — Supplementary Information [file 41467_2021_22401_MOESM1_ESM.pdf]

Supplementary Information

VESPER: Global and Local Cryo-EM Map Alignment Using Local Density Vectors

Xusi Han[1], Genki Terashi[1], Charles Christoffer[2], Siyang Chen[2], and Daisuke Kihara[1,2,*]

[1] Department of Biological Sciences, Purdue University, West Lafayette, Indiana, 47907, USA

[2] Department of Computer Science, Purdue University, West Lafayette, Indiana, 47907, USA

These authors contributed equally to this work: Xusi Han, Genki Terashi

* Corresponding author:

E-mail: dkihara@purdue.edu

(**Supplementary Data 1: Global and Local map search datasets.** Provided as a separate Excel file.)

(**Supplementary Data 2: Comparison with 18 other map alignment scores.** Provided as a separate Excel file.)

The DOT score was compared with 18 other existing scores that were reported in a paper by Joseph AP, Lagerstedt I, Patwardhan A, Topf M, Winn M, "Improved metrics for comparing structures of macromolecular assemblies determined by 3D electron-microscopy". J Struct Biol. 2017, 199(1):12-26.

Those scores are:
Overlap (OVR)
Segment based Manders' Overlap Coefficient, Local cross correlation about zero (SMOC)
Local cross correlation (SCCC)
Cross correlation-coefficient (CCC)
Local mutual information (LMI)
Normalized mutual information (NMI)
Chamfer surface distance score on points selected based on a density threshold range (CDT)
Chamfer surface distance score on points selected using mean filter (CDM)
Chamfer surface distance score on all points at an iso-contour level (CDA)
Normal vector score on surface points selected from a density threshold range (NVT)
Normal vector score on surface points identified by mean filter on binary mask (NVM)
Normal vector score on all points at an iso-contour level (NVA)
Normalized variants of CDT, CDM, & CDA (CDT_GDT), (CDM_GDT), (CDA_GDT)
SMOC, SCCC, LMI are combined with OVR (SMOC_OV), (SCCC_OV), (LMI_OV)
Please refer to the above paper for more explanation of these scores.
These 18 scores were computed with TEMPy (Farabella, I., Vasishtan, D., Joseph, A. P., Pandurangan, A. P., Sahota, H., & Topf, M. (2015). TEMPy: a Python library for assessment of three-dimensional electron microscopy density fits. Journal of applied crystallography, 48: 1314-1323).

The map pairs that were used for the evaluation of the scores were taken from Table 1 of the above paper. From the list, we removed 2 pairs, EMD-1302 (PDB: 2o0f) vs. EMD-1366 (PDB: 1pn6) and EMD-1248 (PDB: 1zo1) vs. 1067 (PDB: 1s1h). The first pair was removed because the associated PDB structures do not overlap with the density maps, and moreover, the structural similarity of the two structures had only marginal TM-Score (0.524 and 0.680, depending on the direction of the comparison), and thus the correct alignment could not be defined. Similarly, the second pair was removed because the first map, the associated PDB entry of EMD-1248 do not overlap with the density map and also TM-scores for the associated structures was very low, 0.066 and 0.362, which are below the commonly used significance score.

The total number of map pairs used were 26, 13 pairs from the "other" category, 5 pairs from ribosome category, and 8 pairs from the virus category. All maps were resampled to the grid

spacing of 3 Å. For each pair, map alignments were constructed by randomly generating normally distributed translational shifts with a standard deviation of 5 Å and uniformly distributed rotational shifts, both relative to the reference alignment. Please refer to the Methods section. For each pair, 100 total alignments were generated.

The 18 scores and the DOT score were evaluated by the accuracy of correct map alignment retrieval. Seven metrics were used. AUC, Area Under the curve; Average precision, the area under the precision-recall curve (i.e. the average of the prevision values at each recall level); Z-score 1 (Z1), the Z-score of the best alignment (i.e. lowest RMSD or log(ALCPS) score) computed from all the alignments; Z-score of top 10 models (Z10), the average Z-score of the 10 best (i.e. lowest RMSD or lowest log(ALCPS)) alignments; #Δlog(ALCPS) and ΔRMSD, the difference of log(ALCPS) and RMSD between the best alignment in the dataset and the first choice by the score; Best ACC, the best accuracy recorded by changing the score cutoff value that was uniformly applied to the all the alignments in the same category; Best F1, the beset F1-score recorded by changing the score cutoff value that was uniformly applied to the all the alignments in the same category. The columns named threshold show the selected score threshold values that were used to compute the best accuracy and the best F1-score.

To compute some of these scores, alignments needed to be classified into "correct" alignments (or more precisely, alignments that have a sufficient closeness to the reference alignments that were defined by superimposing associated PDB structures) and the other incorrect alignments. For log(ALCPS), the cutoff for the Other, Ribosome, and Virus categories were -0.4, 0.82, and -0.5, respectively. These log(ALCPS) cutoff values were taken from the paper by Joseph et al. For RMSD, we used 10 Å as the cutoff.

In the table, the best value for each evaluation metric was highlighted in bold. Row 22 (Best) shows the best value observed for each metric.

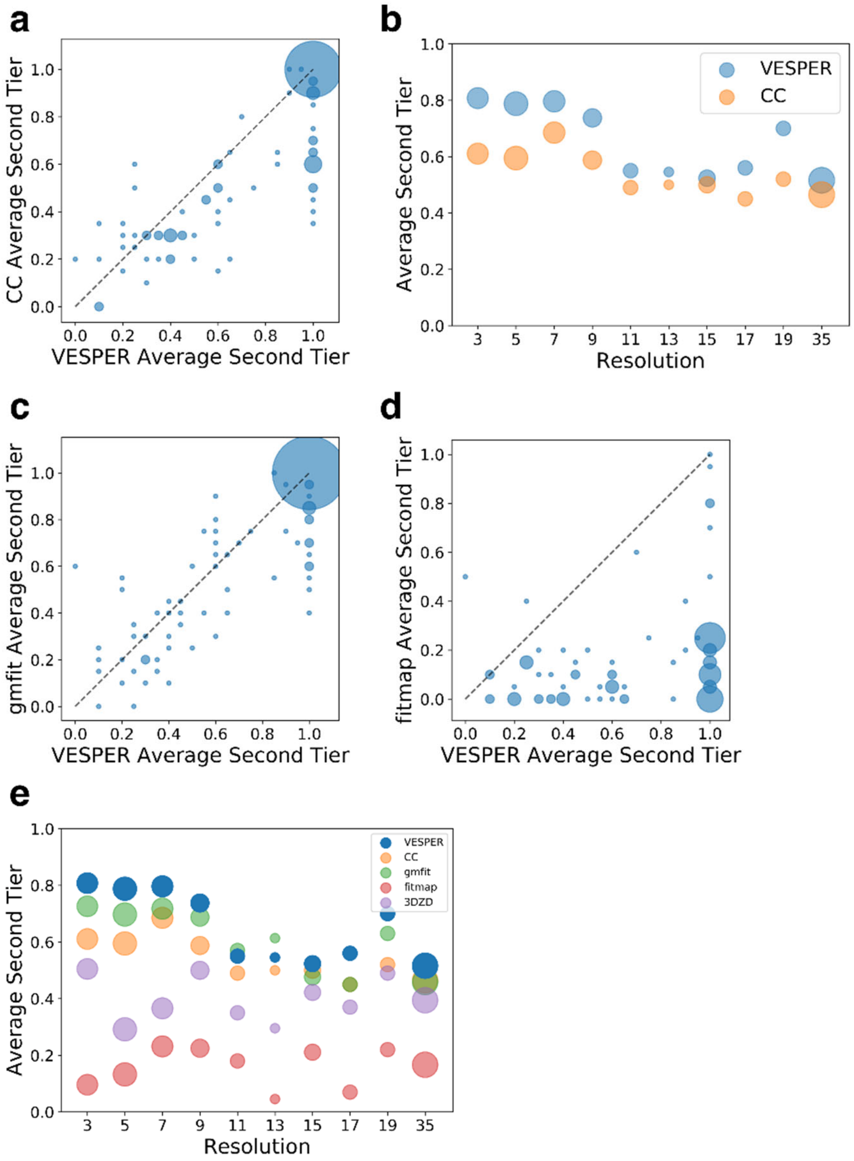

Supplementary Figure 1. Performance of global map search in terms of correct hits within the second tier. Corresponding results considering the first tier are shown in Fig. 2. **a**, The average fraction of correct hits within the second tier for each map group. the x-axis, VESPER with the DOT score; the y-axis, CC. The area of a point is proportional to the number of groups at that data point. **b**, Comparison of VESPER and CC on maps at different resolutions. The average fraction of correct hits within the first tier was considered. Blue, VESPER with the DOT score; orange, CC. **c**, Comparison between VESPER and gmfit in terms of the average fraction of correct maps within the second tier for each map group. **d**, Comparison between VESPER and fitmap in terms of the average fraction of correct maps within the second tier for each map group. **e**, Comparison of the average fraction of correct maps within the second tier at each resolution bin for VESPER (blue), CC (orange), gmfit (green), fitmap (red), and 3DZD (EM-SURFER) (purple).

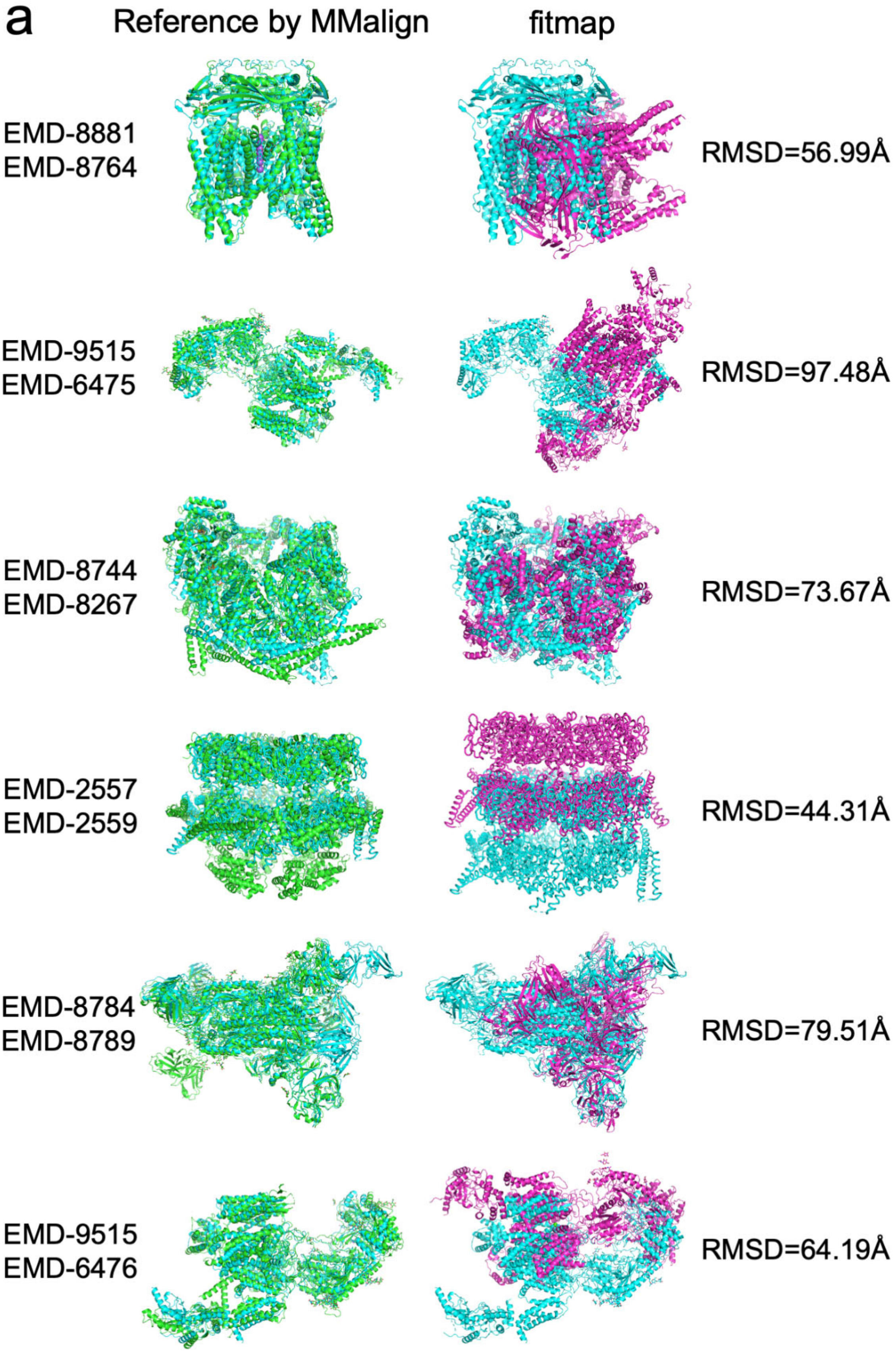
a
Reference by MMalign
fitmap
EMD-8881
EMD-8764
RMSD=56.99Å
EMD-9515
EMD-6475
RMSD=97.48Å
EMD-8744
EMD-8267
RMSD=73.67Å
EMD-2557
EMD-2559
RMSD=44.31Å
EMD-8784
EMD-8789
RMSD=79.51Å
EMD-9515
EMD-6476
RMSD=64.19Å

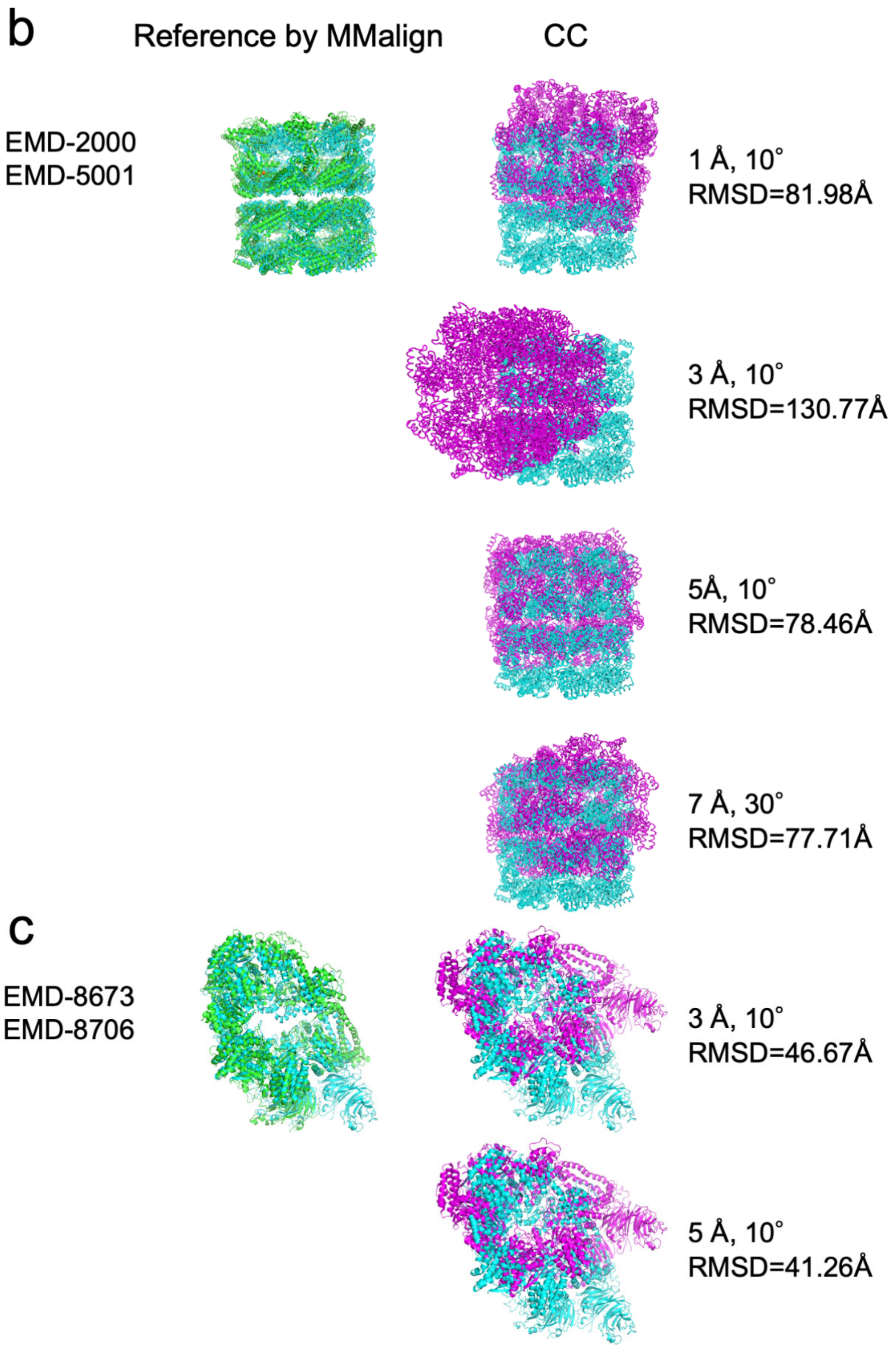

Supplementary Figure 2. Global map alignments with large RMSD values by fitmap and CC in Table 2. This figure shows map alignments in Table 2 that have large RMSD values. Please

refer to Table 2 for other data. **a,** six alignments with large RMSD values by fitmap. The reference alignment with MMalign is shown on the left and the alignment by fitmap is shown on the right. **b,** four alignments with large RMSD values for EMD-2000 with EMD-5001 using CC. **c,** two alignments with large RMSD values for EMD-8673 with EMD-8706. In the second and the third panel, the first alignment is the reference aligned with MMalign.

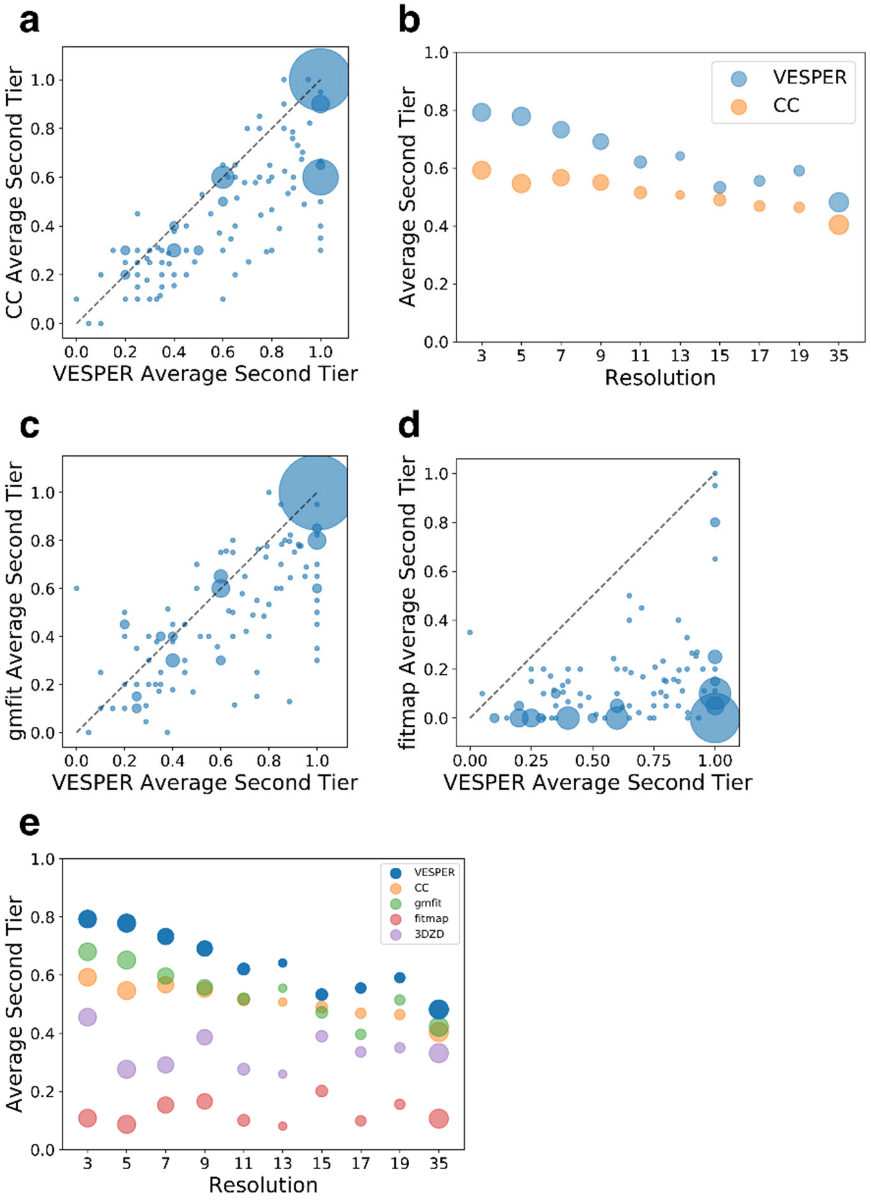

Supplementary Figure 3. Performance of partial map search in terms of correct hits within the second tier. Corresponding results using the first tier are shown in Fig. 3. **a**, the average fraction of correct hits within the second tier for each map group. the x-axis, VESPER with the DOT score; the y-axis, CC. **b**, Comparison of VESPER and CC on maps at different resolutions. The average fraction of correct hits within the first tier was considered. Blue, VESPER with the DOT score; orange, CC. **c**, Comparison between VESPER and gmfit in terms of the average fraction of correct maps within the second tier for each map group. **d**, Comparison between VESPER and fitmap in terms of the average fraction of correct maps within the second tier for each map group. **e**, Comparison of the average fraction of correct maps within the second tier at each resolution bin for VESPER (blue), CC (orange), gmfit (green), fitmap (red), and 3DZD (EM-SURFER) (purple).

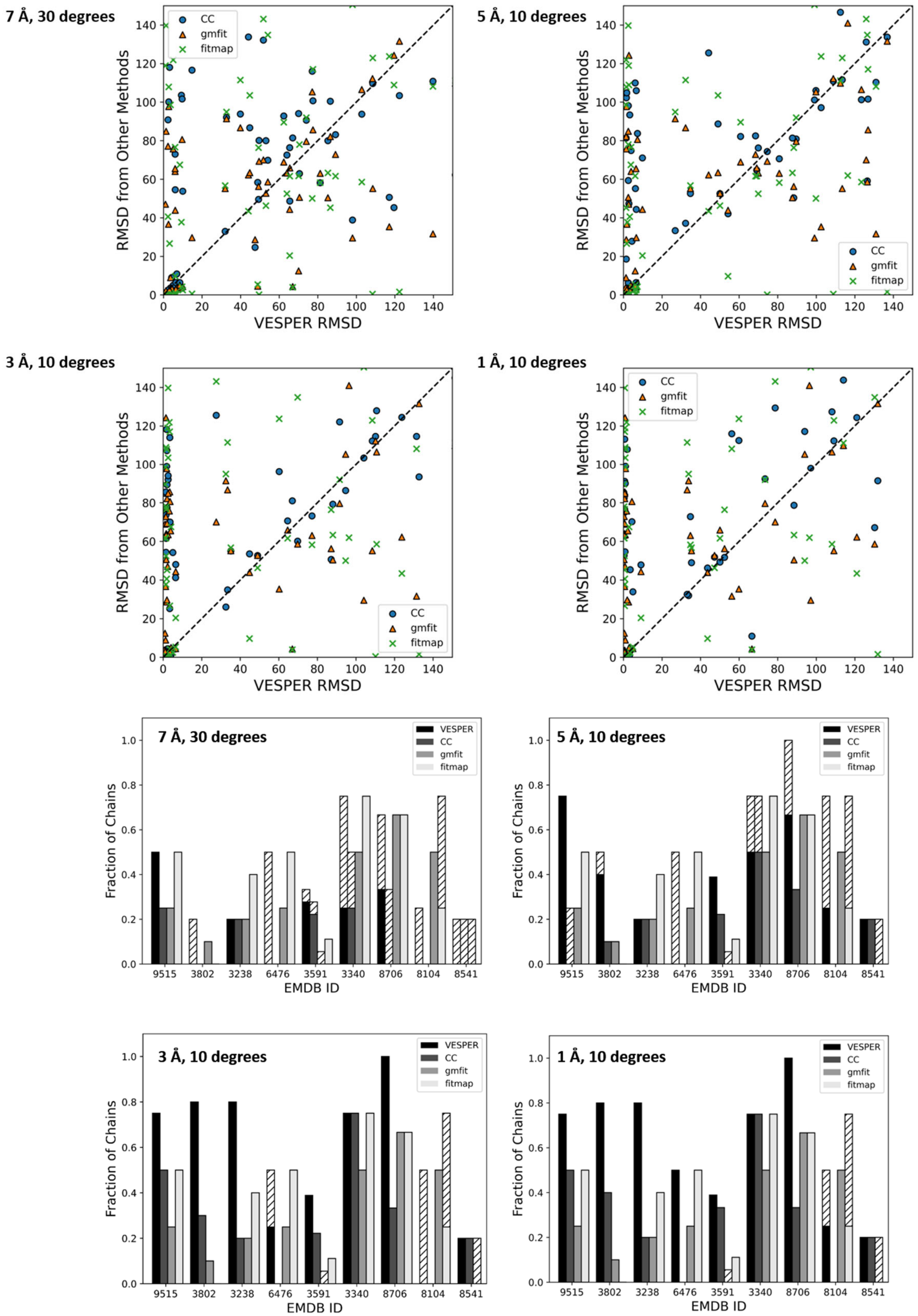
7 Å, 30 degrees
5 Å, 10 degrees
3 Å, 10 degrees
1 Å, 10 degrees
RMSD from Other Methods
VESPER RMSD
CC
gmfit
fitmap
7 Å, 30 degrees
5 Å, 10 degrees
3 Å, 10 degrees
1 Å, 10 degrees
Fraction of Chains
EMDB ID
VESPER
CC
gmfit
fitmap
9515
3802
3238
6476
3591
3340
8706
8104
8541

Supplementary Figure 4. Performance of partial map alignment (atomic model fitting). Four different parameter combination were used: voxel spacing of 7 Å with rotation interval of 30 degrees, 5 Å and 10 degrees, 3 Å and 10 degrees, and 1 Å and 10 degrees.

Top four panels, comparison of RMSD of the top-scoring map alignment by VESPER (DOT) with CC, gmfit, and fitmap. EM maps in the dataset are listed in Table 3. Blue circles, comparison against CC; orange triangles, gmfit; green crosses, fitmap, respectively. Lower panels, the fraction of query chains for each map that had the top-scoring alignment with an RMSD of 5.0 Å or less (solid gray bars) and 10.0 Å or less (including hatched bars). Black bars, VESPER (DOT); dark gray, CC; medium gray, gmfit; pale gray, fitmap.

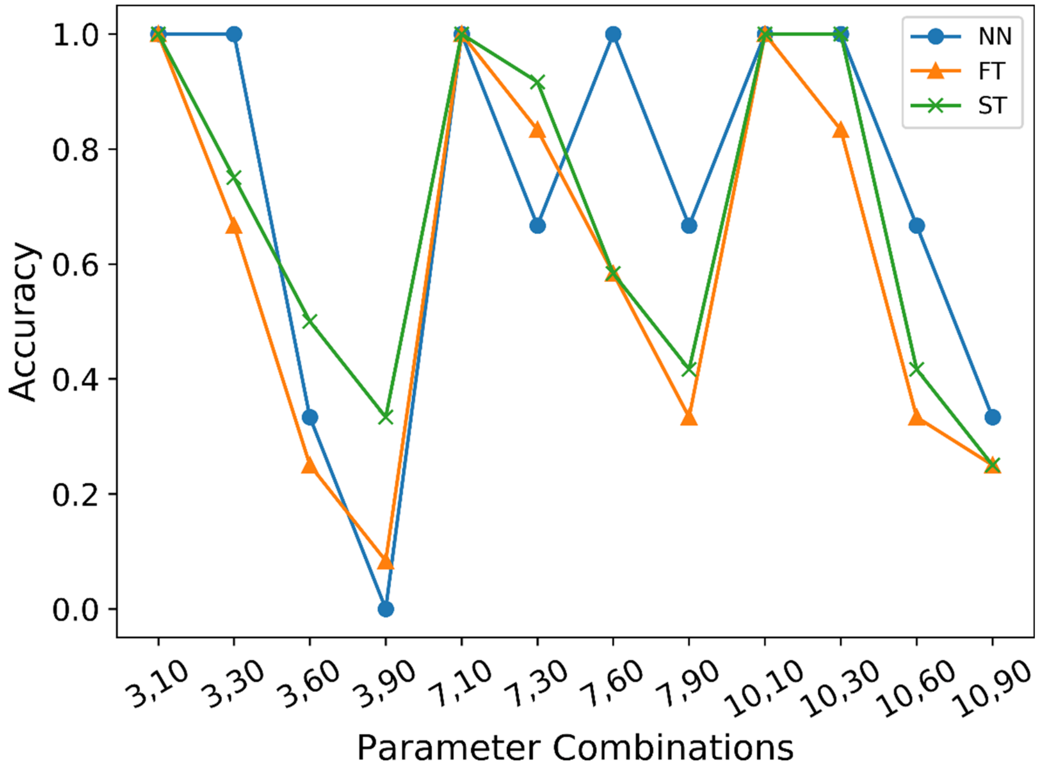

Supplementary Figure 5. Global map retrieval performance using different voxel and angle spacing combinations. The average fraction of correct maps within the nearest neighbor (NN, i.e. top hit, blue), within the first tier (FT, orange), and the second tier (ST, green) for three query maps, EMD-3661, EMD-8724 and EMD-1203, were plotted. Along the x-axis, combinations of (voxel, angle) spacing values used are shown.

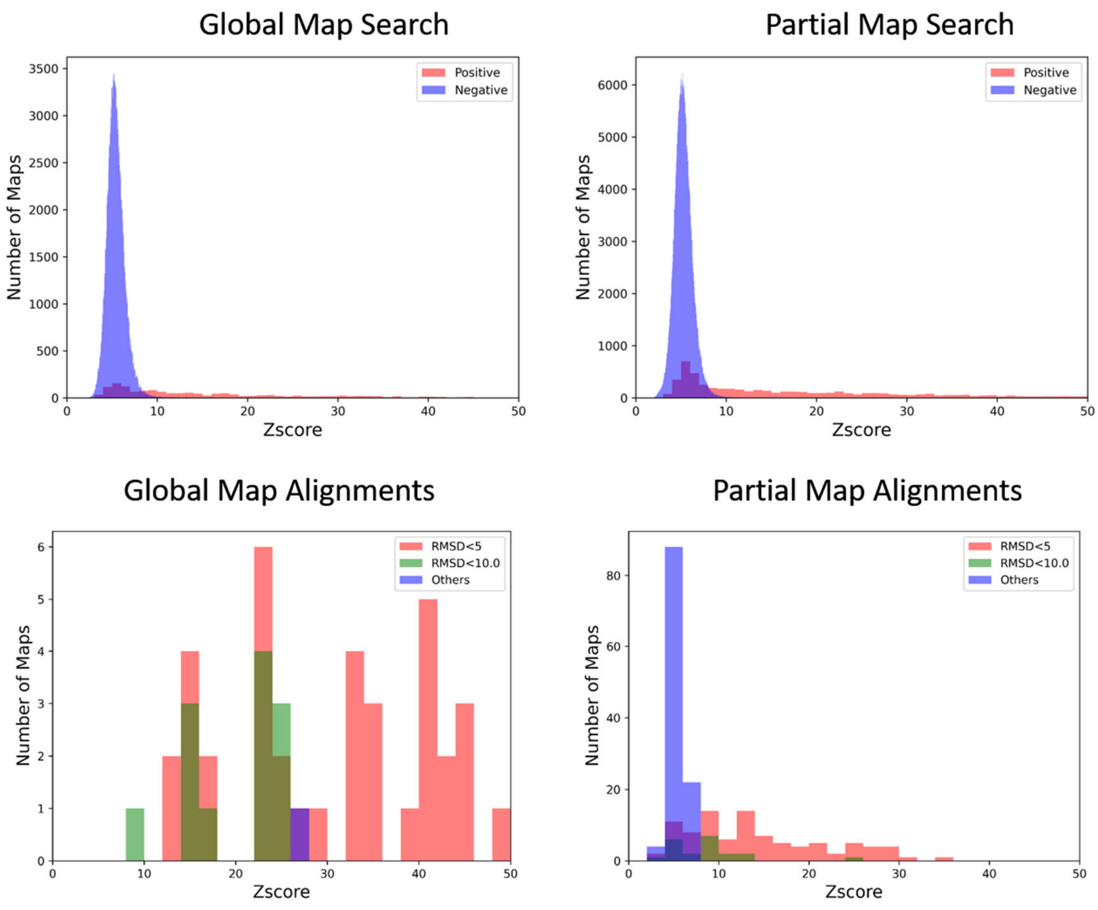

Supplementary Figure 6. Z-score distributions of map search and alignment.

The top left panel shows the Z-score distribution of correct maps (maps that are in the same group as the query used) and incorrect maps in the global map search (reported in Figure 2). The top right panel is the distribution of the partial map search (Figure 3) results. The correct maps are those in the same class as the query.

The bottom left is the distribution of the z-score of global map alignments. Data are from Table 2, where 15 pairs of maps were aligned with VESPER (DOT) using 4 different translation and angle parameter combinations. Alignments within 5 Å, 10 Å, or larger are shown in red, green, and blue bars. The bottom right is the distribution for the partial map alignments with simulated maps of protein chains against the experimental EM maps. The dataset of chains is shown in Table 3. For each chain, the results of the top z-score alignment using four different parameter combinations were used to plot.

Judging from these distributions, a Z-score of 10 would be a proper cutoff for distinguishing correct and incorrect global and partial map retrieval and partial map alignments (i.e. chain structure fitting) of better than a 5 Å RMSD. The data for the global map alignments (bottom left) includes only one incorrect (others) cases, thus a proper cutoff could not be determined.

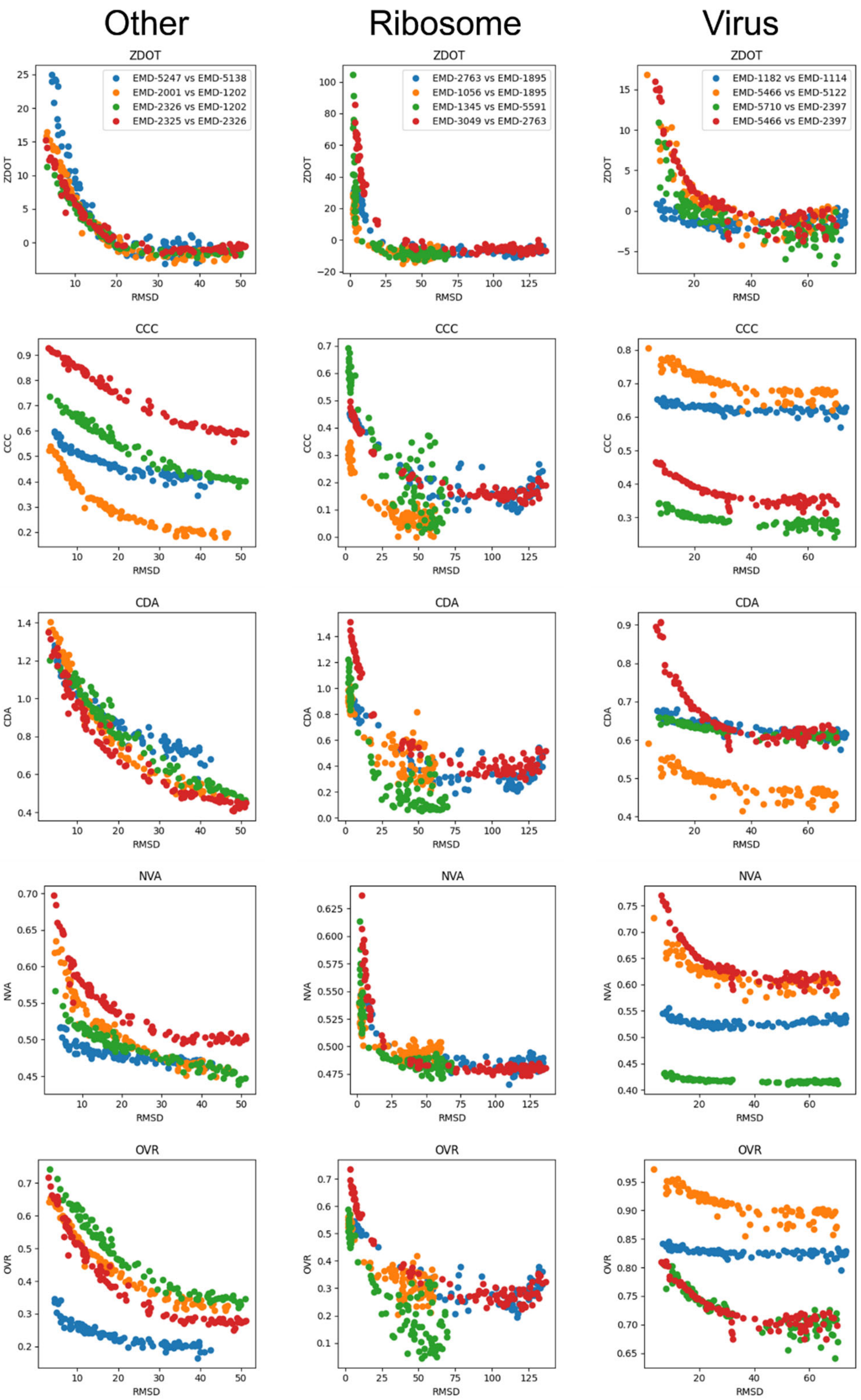

Supplementary Figure 7. Correlation of RMSD with six scores.

RMSD values were compared with DOT Z-score (ZDOT), cross correlation-coefficient (CCC), Chamfer surface distance score on all points at an iso-contour level (CDA), normal vector score on all points at an iso-contour level (NVA), and overlap (OVR). These scores were selected because they represent different types of scores. For each score comparison, four map pairs were used from the three categories, others, ribosome, and virus maps. The four pairs are shown in different colors in each panel.
