# Supplementary material for: VESPER: global and local cryo-EM map alignment using local density vectors
Source: Nat Commun. 2021 Apr 7;12:2090. doi: 10.1038/s41467-021-22401-y (PMC8027200; doi:10.1038/s41467-021-22401-y)
Supplement: Supplementary file 3 — Description of Additional Supplementary Files [file 41467_2021_22401_MOESM3_ESM.pdf]

Description of Additional Supplementary Files

Supplementary Data 1: Global and Local map search datasets

Supplementary Data 2: Comparison with 18 other map alignment scores. The DOT score was compared with 18 other existing scores that were reported in a paper by Joseph AP, Lagerstedt I, Patwardhan A, Topf M, Winn M, “Improved metrics for comparing structures of macromolecular assemblies determined by 3D electron-microscopy”. J Struct Biol. 2017, 199(1):12-26.
